# Jia-Wei-Kai-Xin-San, an Herbal Medicine Formula, Ameliorates Cognitive Deficits via Modulating Metabolism of Beta Amyloid Protein and Neurotrophic Factors in Hippocampus of Aβ_1-42_ Induced Cognitive Deficit Mice

**DOI:** 10.3389/fphar.2019.00258

**Published:** 2019-03-19

**Authors:** Yue Zhu, Yiwei Shi, Cheng Cao, Zhenxiang Han, Mengqiu Liu, Mingzhu Qi, Renjie Huang, Ziqiang Zhu, Dawei Qian, Jin-ao Duan

**Affiliations:** ^1^Jiangsu Key Laboratory for High Technology Research of TCM Formulae and Jiangsu Collaborative Innovation Center of Chinese Medicinal Resources Industrialization, Nanjing University of Chinese Medicine, Nanjing, China; ^2^Department of Neurology and Rehabilitation, Shanghai Seventh People’s Hospital, Shanghai University of Traditional Chinese Medicine, Shanghai, China

**Keywords:** Alzheimer’s disease, beta amyloid protein, neurotrophic factor, Jia-Wei-Kai-Xin-San, Chinese medicine formula

## Abstract

Jia-Wei-Kai-Xin-San (JWKXS) is a Chinese medicine formula applied for treating morbid forgetfulness in ancient China. Today, this formula is frequently applied for Alzheimer’s disease and vascular dementia (VD) in clinic. Here, we developed it as granules and aimed to evaluate its anti-AD effect on β amyloid protein 1–42 (Aβ_1-42_) induced cognitive deficit mice and reveal the possible molecular mechanisms. Firstly, daily intra-gastric administration of chemically standardized of JWKXS granules for 7 days significantly ameliorated the cognitive deficit symptoms and inhibited cell apoptosis in hippocampus on Aβ_1-42_ injection mice. JWKXS granules significantly decreased Aβ level, increased superoxide dismutase activity and decreased malondialdehyde level in hippocampus of model mice. It also restored acetylcholine amounts, inhibited acetylcholinesterase activities and increased choline acetyltransferase activities. In addition, JWKXS granules enabled the transformation of precursors of NGF and BDNF into mature forms. Furthermore, JWKXS granules could regulate gene expressions related to Aβ production, transportation, degradation and neurotrophic factor transformation, which led to down-regulation of Aβ and up-regulation of NGF and BDNF. These findings suggested that JWKXS granules ameliorated cognitive deficit via decreasing Aβ levels, protecting neuron from oxidation damages and nourishing neuron, which could serve as alternative medicine for patients suffering from AD.

## Introduction

Alzheimer’s disease is the highest incidence dementia in aging society. According to epidemiological calculation, it is estimated that 40 million people have dementia worldwide and most of them are older than 60 years. According to a report made by Alzheimer’s Association, AD has become the sixth leading cause of death in the United States and this figure is estimated to be double every 20 years, until at least 2050 (Alzheimer’s Association, 2018). During over 100 years since AD firstly described in 1906, it has been realized AD is caused by genetic abnormalities combined with multiple factors. As to genetic cause, carrying *ApoE4* gene and gene mutations of amyloid precursor protein (APP), presenilins 1 and 2 greatly enhance AD susceptibility. In addition to genetic mutation, pathological factors including vascular disease, diabetes, obesity, depression greatly enhance AD risk. Not limited to the genetic and pathological factors, low education degree and unhealthy life style such as mental, physical inactivity, and smoking also increase AD susceptibility (Scheltens et al., 2016).

β-Amyloid peptide (Aβ) hypothesis is the most famous pathological hypothesis of AD. Aβ is not only the pathological hallmark required for AD diagnosis but also exerts obvious neurotoxicity no matter *in vivo* or *in vitro*. Therefore, researchers spend large energies and costs on screening small molecules or trying to develop antibodies aiming at suppressing Aβ production or removing Aβ. Unfortunately, most of drug candidates fail in clinic research though exerting potency on animal models. Now, it has been accepted that AD is a complex disease combined with multiple pathological factors rather than single cause. Dysfunction of cholinergic neuron transduction, despaired anti-injury ability, over stimulation of neuronal inflammation and impaired neuron nourishment all contribute to the initiation and progress of AD. Therefore, single compound or antibody only aiming at degrading Aβ seems unable to resolve these pathological problems simultaneously.

Traditional Chinese medicine (TCM) has accumulated abundant experiences in treating cognitive decline and dementia by using formulae. TCM formulae are composed of several medicinal herbs, which are characterized by multiple compounds and action targets. In TCM theory, cause of dementia ascribes to spleen asthenia and phlegm accumulation, deficits of heart-qi and heart-yin. Therefore, Chinese medicine formulae for AD treatment are usually created and applied for invigorating spleen to remove phlegm and nourishing heart-qi and heart-yin. Based on this scenario, Jia-Wei-Kai-Xin-San (JWKXS) was described by Bo-xiong Fei (1800∼1879) in Qing dynasty as an effective formula for treating amnesia, which is also applied for AD treatment nowadays. JWKXS is composed of Ginseng Radix et Rhizoma, Polygalae Radix, Acori Tatarinowii Rhizoma, Poria, Ophiopogonis Radix, Schisandra Chinensis Fructus, Curcumae Radix, Gardeniae Fructus. This formula is created based on two famous formulae: Kai-Xin-San and Sheng-Mai-San. Kai-Xin-San, composed of Ginseng Radix et Rhizoma, Polygalae Radix, Acori Tatarinowii Rhizoma and Poria, possesses the function of replenishing Xin-qi and strengthening the spleen, inducing resuscitation and eliminating water-dampness. Modern researches imply that Kai-Xin-San ameliorates cognitive decline by decreasing Aβ production, protects neuron from oxidative stress and improve long-term potentiation (Nishiyama et al., 1994; Zhang et al., 1994; Lu et al., 2017). Sheng-Mai-San, composed of Ginseng Radix et Rhizoma, Ophiopogonis Radix and Schisandra Chinensis Fructus, possesses the function of benefiting Xin-qi and promoting the production of body fluid, astringing heart-yin and arresting perspiration. According to reports, Sheng-Mai-San protects brain from ischemia by decreasing lipid, increasing cerebral blood supply and improving blood rheology. This formula also improves memory and learning ability on transgenic AD mice and *C. elegans* (Wang et al., 2010; Li et al., 2013). Although Kai-Xin-San and Shen-Mai-San both improve cognitive decline on animal studies, single use of either one formula cannot satisfy nourishing heart-qi and heart-yin simultaneously. Therefore, these two formulae are seldom single used while frequently combined and added with other herbs, which is more suitable for the pathology and treatment of AD. In addition, Curcuma Radix promoting flow of qi and dispersing the stagnated qi and Gardeniae Fructus removing dampness and heat are added in JWKXS to strengthen the efficacy. Though the Anti-AD potential and related components of medicinal herbs in JWKXS are frequently reported (Sun et al., 2013; Wang et al., 2016), the action mechanisms of JWKXS formula have never been explored and reported, which greatly hinders the drug development and clinical use of this potent anti-AD formula. To develop JWKXS as an anti-AD drug, we made this formula into granules and evaluated its effects on learning and memory on an Aβ induced cognitive deficit mice. Afterward, the effects of JWKXS granules on Aβ production, oxidative injury, cholinergic neuron, and neurotrophic factor transformation were evaluated and the possible action targets were explored.

## Materials and Methods

### Herbal Materials

Ginseng Radix et Rhizoma, Polygalae Radix, Acori Tatarinowii Rhizoma, Poria, Ophiopogonis Radix, Schisandra chinensis Fructus, Curcuma Radix and Gardeniae Fructus, were purchased from Suzhou Tianling Chinese Herbal Medicine, Co., Ltd. and identified as qualified medicines by one of co-authors professor Jin-ao DUAN. The botanical, herbal and Chinese name of the corresponding herb were listed in Supplementary Figure S1. The quality of the herbs and herbal extracts was consistent with the standards of Chinese Pharmacopoeia (2015).

### Preparation of JWKXS Granules

The eight component herbs, Ginseng Radix et Rhizoma (150 g), Polygalae Radix (100 g), Acori Tatarinowii Rhizoma (100 g), Poria (150 g), Ophiopogonis Radix (150 g), Schisandra chinensis Fructus (100 g), Curcuma Radix (60 g) and Gardeniae Fructus (100 g), were soaked in 60% ethanol (1:8 w/v) for 1 h and extracted twice by refluxing for 2 h. All the filtrates were combined and concentrated under reduced pressure below 70°C to receive a density of 1.8 g crude drug per milliliter. The condensed extracts were mixed with dextrin and sugar powder to make up JWKXS granules. The granules were stored in the refrigerator at 4°C.

The contents of geniposide (derived from Gardeniae Fructus), polygalaxanthone III and 3, 6′-disinapoyl sucrose (derived from Polygalae Radix), α-asarone andβ-asarone (derived from Acori Tatarinowii Rhizoma), deoxyschizandrin (derived from Schisandra chinensis Fructus) of JWKXS extracts were 1035.61, 283.72, 260.15, 10.13, 109.47, 152.9 μg/g by UPLC-DAD analysis. The contents of ginsenoside Rg_1_, ginsenoside Rb_1_ (derived from Ginseng Radix et Rhizoma) and Ophiopogonin D (derived from Ophiopogonis Radix) of JWKXS granules were 133.3, 83.4, and 36.7 μg/g by HPLC-ELSD analysis. The representative chromatograms were listed in Supplementary Figures S2A,B. All of the chemical markers were purchased from Sichuan Victory Biotechnology, Co., Ltd. (Chengdu, China) and the purities were higher than 98% by normalization of peak areas detected by HPLC–DAD.

### Animals and House Conditions

Male ICR mice weighing 20–22 g were obtained from the Laboratory Animal Services Center, Nanjing University of Chinese Medicine. Animals were maintained on a 12 h light/dark cycle (lights on at 6:00 a.m., lights off at 6:00 p.m.) under controlled temperature (22 ± 2°C) and humidity (50 ± 10%), and were given standard diet and water *ad libitum*. They were allowed to acclimatize for 7 days before model development. The experiments on animals have been approved by the Animal Experimentation Ethics Committee of Nanjing University of Chinese Medicine and conformed to the guidelines of the “Principles of Laboratory Animal Care” (NIH Publication No. 80-23, revised 1996). Efforts were made to minimize the number and suffering of the animals.

### Development of Aβ Induced Cognitive Deficit Mice and Drug Treatment

Aβ_1-42_ peptides at final concentration 5 μg/μL were dissolved in saline and incubated for 7 days at 37°C for aging. Then 50 μl sample was mixed with 150 μl thioflavin solution (5 μM in 50 mM glycine, pH 8.5) to determine Aβ aggregation by measuring the fluorescence intensity at emission 435 nm and excitation 480 nm, which showed that Aβ fragment had aggregated. Mice were separated into five groups (*n* = 10 each). Control groups mice were raised normally. For model group mice, 5 μL aging Aβ were transcranial bilateral injected in hippocampus [anterior–posterior (AP) = -2.3 mm, medial–lateral (ML) = ± 1.8 mm from the bregma and dorsal–ventral (DV) = 2.0 mm from cerebral dura mater, which were standardized from the stereotaxic atlas of Paxinos and Watson] (5 μL for right-side). Sham group animals were subjected to the same surgical procedure as model group animals with saline instead of Aβ. Model group mice were recovered for 14 days and then some of them were orally treated with JWKXS granules at two dosages. JWKXS extracts were treated at low and high dosages. Low dosage treatment was set as 1.5 g/kg/day (converted to crude herbal materials at 9 g/kg/day) and high dosage treatment was set as 3 g/kg/day (converted to crude herbal materials at 18 g/kg/day). The dosage of JWKXS treatment was set according to the clinical usage and dosage of each herb in formula also conform to corresponding dosage stipulation in Chinese pharmacopeia (2015 edition). Huperzine A was set as positive control at dosage of 0.05 μg/kg/day. For mechanism studies, RAGE antagonist FPS-ZM1 (Millipore, MA) was treated at 1 μg/kg/d (i.p.); LRP1 antagonist receptor-associated protein (RAP) was treated at 1 μg/μL (hippocampus injection at 5 μL); tPA antagonist tPA-stop (American Diagnostica, Inc., Stamford, CT, United States) was dissolved in 5 μL artificial CSF and transcranial bilateral injected in hippocampus at 1.5 μg/kg; MMP-9 antagonist JAJN966 was treated at 10 μg/kg/d (i.p.).

### Morris Water Maze Test

The learning and memory abilities were evaluated by the Morris water maze test. The experimental apparatus (Water Mazes, TSE Systems, China) consisted of a blank circular water basin (100 cm in diameter, 35 cm in height), containing water (23 ± 1°C) to a depth of 15.5 cm, which was rendered opaque by adding black ink. Four poles along the circumference of the basin divided the basin into four equal quadrants and various prominent visual cues (e.g., pictures, lamps, etc.) were located on the inner wall. A round platform (4.5 cm in diameter, 14.5 cm in height) was immersed 1 cm below the water surface and fixed in one quadrant. Before tests, each mouse received training. In one training session, the mouse was put in one quadrant facing the wall at one of three randomized starting positions (in three different quadrants without the platform). Then the mouse was given a 60 s free swimming, and then helped to climb on the platform and rested for another 20 s. If the mouse failed to find the platform within 60 s, the mouse would be guided to find the platform and allowed to stay on the platform for another 20 s. This training lasted four consecutive days and the mouse was placed in different starting position of quadrant in each day. In normal test, the mouse was put in one quadrant facing the wall and given 60 s to find the platform. The time and routine to find the platform were recorded and this test was termed as spatial test. Each mouse performed four tests per day, and the inter-trial interval was 60 s. Twenty hours after the spatial test, the platform was removed and the mouse was allowed to swim freely in the water basin and the time to swim in the target quadrant (where the removing platform previous placed) and the number of times crossing over the platform site were recorded.

### TUNEL Assay for Apoptosis in Hippocampal Cells

After drug treatment, mice were anesthetized and perfused through the heart with normal saline and 4% paraformaldehyde sequentially. Mice were decapitated and the brains were removed and fixed in 4% paraformaldehyde for observation of neuronal cell apoptosis in hippocampus by terminal deoxyribonucleotidyl transferase-mediated dUTP nick end labeling (TUNEL) assay. TUNEL assay was performed using the TdT-FragELTM DNA Fragmentation Detection Kit (Merck, Germany) according to the manufacture instruction.

### ELISA Assays

After water maze tests, the mice were sacrificed and the hippocampus were removed and frozen in liquid nitrogen and kept in -80°C until assay. Hippocampus tissues were homogenized in sodium phosphate buffer (0.1 M PBS, pH 7.4) containing a protease inhibitor cocktail (Roche). Lysates were centrifuged 15,000 × *g* for 30 min at 4°C. The supernatant was separated and protein levels were determined by commercial ELISA kits according to each manufacturer’s instruction. Aβ amounts were determined by amyloid beta protein mouse ELISA Kit (Jinyibai Company, China). proNGF and NGF levels were determined by mouse proNGF ELISA kit and mouse NGF ELISA kit (CUSABIO, Houston, TX, United States). proBDNF and BDNF levels were determined by mouse proBDNF ELISA kit and mouse BDNF ELISA kit (Aviscera Bioscience, Santa Clara, CA, United States). The protein content was expressed as ng/g wet weight of tissue.

### Determination of SOD Activity and MDA Analysis

Superoxide dismutase (SOD) activity was determined by SOD activity assay kit (BioVision, Mountain View, CA, United States) according to the manufacturer’s protocol. Hippocampus tissue homogenates were prepared same as that for Aβ assays. Briefly, 20 μl of hippocampus homogenate was added with 200 μl of water soluble tetrazolium working solution and 20 μl enzyme working solution and incubated at 37°C for 20 min in a 96-well plate. The absorbance of each well was measured at 450 nm wavelength using a microplate reader (Enspire, Perkin-Elmer).

Malondialdehyde (MDA) level of hippocampus was determined according to reported method (Xian et al., 2014b). In brief, 100 μl of hippocampus homogenate was mixed with 1.5 ml of 20% (v/v) acetic acid, 1.5 ml of 0.8% (w/v) thiobarbituric acid, 200 μl of 8% (w/v) sodium dodecyl sulfate and heated for 60 min at 95°C and cooled to room temperature. The mixture was extracted with 5 ml *n*-butanol and then centrifuged at 3000 *g* for 10 min. The *n*-butanol layer was collected and measured at 532 nm using a microplate reader (Enspire, Perkin-Elmer).

### Determination Amounts of Ach and Activities of AChE and ChAT

Hippocampus tissue homogenates were prepared same as that for Aβ assays. The Ach level in hippocampus was measured according to described method and modified (Xian et al., 2015). Briefly, 0.5 μl of tissue homogenate was added sequentially with 1.5 ml of distilled water, 0.2 ml of calabarine sulfate (1.54 mmol/l), 0.8 ml of trichloroacetic acid (1.84 mol/L) and then centrifuged at 3000 *g* for 15 min. 1 ml of supernatant was mixed with 1.0 ml of alkaline hydroxylamine solution (prepared by mixing equal volumes of 3.5 M sodium hydroxide and 2 M hydroxylamine hydrochloride solutions) and incubated at room temperature for 15 min. Afterward, 0.5 ml of hydrochloride acid (4 mol/l) and 0.5 ml of ferric chloride (0.37 mol/l) were added and the mixtures were measured at 540 nm using a microplate reader (Enspire, Perkin-Elmer).

The activity of acetylcholinesterase (AChE) was determined according to a described method (Xie et al., 2010). Briefly, the reaction mixture consisted of 1 μl of hippocampus homogenate, 399 μl of phosphate buffer (Na_2_HPO_4_ 200 mM) and incubated 30 min in room temperature. 80 μl mixture were added with 98 μl H_2_O and 2 μl iso-OMPA (10 mM) in 96 well plate and incubated 15 min. Then the mixture were added with 10 μl dithiobisnitrobenzoic acid (DTNB, 10 mM) and acetylthiocholine (Ach, 12.5 mM) and kept avoid of light. The absorbance of mixture were determined at 410 nm for 10 min using a microplate reader (Enspire, Perkin-Elmer).

Choline acetyltransferase (ChAT) activities were determined using the assay kit (Nanjing Jiancheng Bioengineering Company, China). The absorbance at 324 nm were determined using a microplate reader (Enspire, Perkin-Elmer).

### Western Blot Analysis

The details of SDS-PAGE and western blot analysis could be referred to our previous published work (Xie et al., 2010). In brief, the total protein samples were loaded and separated by 15% sodium dodecyl sulfate-polyacrylamide gel electrophoresis (SDS-PAGE) and then transferred to nitrocellulose membrane. The membranes were blocked with 5% BSA, washed four times with Tris-buffered saline plus Tween (TBS-T, 15 min each time), and then incubated with the following primary antibodies: rabbit polyclonal anti-NGF (H-20, 1:1000, Santa Cruz Biotechnology), rabbit polyclonal anti-BDNF (N-20, 1:1000, Santa Cruz Biotechnology) mouse monoclonal anti-tubulin (T-6074, 1:10000, Sigma-Aldrich) at 4°C temperature overnight. After overnight incubation at 4°C, the membranes were washed four times with TBS-T and then incubated with HRP conjugated secondary antibodies according to each species for another 2 h at room temperature. The relative band density was determined by using the Bio-Rad Imaging System (Hercules, CA, United States) with an enhanced chemiluminescence (ECL) western blotting substrate kit (Tiananmen, China). Gel documentation and relative quantification of protein bands were performed with Image-J Digital Imaging System.

### Real-Time Quantitative PCR

To quantify the mRNA expression levels of the regulation genes of Aβ and neurotrophic factor, total RNA from brain tissues were isolated by Trizol reagent (Invitrogen, Carlsbad, CA, United States) according to the manufacture’s instruction. 1 μg of total RNAs extracted from hippocampus were reverse transcribed by using PrimeScript^TM^ RT reagent Kit with gDNA Eraser (Takara, Japan) according to the manufacture’s instruction, and the cDNA of each sample was obtained. Real-time quantitative PCR was performed on equal amounts of cDNA by using SYBR Green Master mix with Rox reference dye (Roche, Germany). The SYBR green signal was detected by Applied Biosystems 7500 fast real-time PCR system. Transcript levels were quantified by using the ΔΔCt value method. The specificity of PCR amplification product was confirmed by the melting curves and gel electrophoresis. The primers for genes accounting for Aβ metabolism were as follows: 5′-CAG TGA GCC CAG AAT CAG C-3′ (sense primer, S) and 5′-CAG AGT CCA CCC CAA AAG G-3′ [anti-sense primer (AS)] for APP (145 bp; BC070409.1); 5′-CAA GGC CTC ATC ATG TGT TC-3′ (S) and 5′-GTC ATA ACC TGG GAC CGG-3′ (AS) for BACE1 (143 bp; BC048189.1); 5′-CAT CCA CTG GAA AGG CC-3′ (S) and 5′-ATT ACG CTA TGC ACC TCA GAG-3′ (AS) for presenilin 1 (147 bp; NM_008943.2); 5′-CAT CTA CCT CGG GGA AGT G-3′ (S) and 5′-CAC TCC GGC AGG TAC TTG-3′ (AS) for presenilin 2 (195 bp; BC010403.1); 5′- ACC CTG AGA CGG GAC TCT T-3′ (S) and 5′-CTG ACT CGG AGT TGG ATG G-3′ (AS) for RAGE (164 bp; BC061182.1); 5′-GAA GAC CCC GAG CAC ATG A-3′ (S) and 5′-CAC AGC TGT TGG TGT CGT TG-3′ (AS) for LRP1 (154 bp; NM_001130490.1); 5′-CTG GAG GTC AAT GGG AAG TC-3′ (S) and 5′-ACC GTC TCC ATG TTG CAG T-3′ (AS) for NEP (153 bp; NM_001289462.1); 5′-AAT GTG CGG GTG GCA ATA G-3′ (S) and 5′-GGT CAG CAT TTT GCC ATT TC-3′ (AS) for IDE (141 bp; NM_031156.3); 5′-AAC GGA TTT GGC CGT ATT GG-3′ (S) and 5′-CTT CCC GTT CAG CTC TGG G-3′ (AS) for GAPDH (195 bp; AF106860.2). The primers for genes accounting for neurotrophic factor metabolism were as follows: 5′-GAC TCA AGG GAC TTT CGG TG-3′ (S) and 5′-CTC GAA GCA AAC CAG AGG TC-3′ (AS) for plasminogen (188 bp; NM_008877.3); 5′-TGA CAA CGA CAT CGC ATT AC-3′ (S) and 5′-TTC AGC CGG TCA GAG AAG-3′ (AS) for tPA (186 bp; NM_008872.3); 5′-GCC TCT TCC ACA AGT CTG-3′ (S) and 5′-TCA AAG GGT GCA GCG ATG-3′ (AS) for PAI (167 bp; M33960.1); 5′-TTG GCC CTC ATC AAT GCT GTA-3′ (S) and 5′-AGA TAC CAC CAG CCT CAT TGG-3′ (AS) for neuroserpin (184 bp; AJ001700.1); 5′-CTT TGA GGA TCC GCA GAC C-3′ (S) and 5′-CTG ACG TGG GTT ACC TCT G-3′ (AS) for MMP9 (132 bp; BC046991.1); 5′-CTT TGA GGA TCC GCA GAC C-3′ (S) and 5′-AGC TGT TGC TGA CAA GAT GGT GGT-3′ (AS) for TIMP-1 (180 bp; U54984.1); 5′-AAC GGA TTT GGC CGT ATT GG-3′ (S) and 5′-CTT CCC GTT CAG CTC TGG G-3′ (AS) for GAPDH (195 bp; AF106860.2).

### Data Analysis

Multiple comparisons were made by using one-way ANOVA followed by a Bonferroni *post hoc* analysis if appropriate (version 13.0, SPSS, IBM, Corp., Armonk, NY, United States). Before ANOVA analysis, normal distribution test was carried out firstly. The control group was varied in different experiments, and which was specified in the figure legends. Data were expressed as Mean ± SEM, where *n* = 5–8. Statistically significant changes were classed as significant [^∗^] where *p* < 0.05, highly significant [^∗∗^] where *p* < 0.01.

## Results

### JWKXS Treatment Ameliorated Cognitive Deficits in Aβ-Injected Mice and Prevented Cell Apoptosis in Hippocampus

**FIGURE 1:**
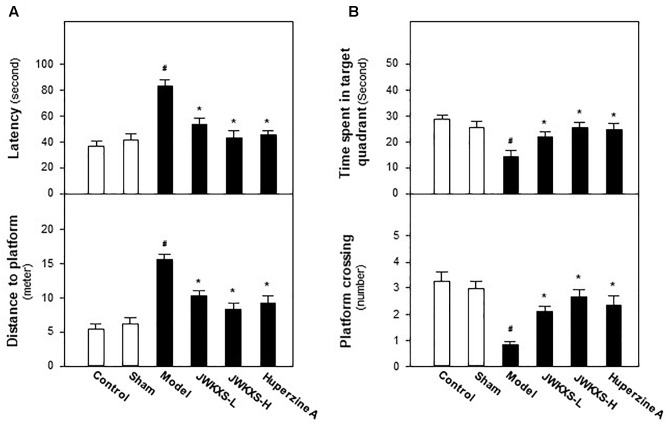
Jia-Wei-Kai-Xin-San ameliorated cognitive deficits on Aβ injection mice. **(A)** After 7 days of JWKXS treatment, all groups of mice were employed for water mazing test. A platform was located in one quadrant of the water basin. The time for mice to find and climb on the platform was termed as latency. A camera and related software recorded and calculated the routine before mice climbing on the platform. **(B)** After the test described in **(A)**, the platform was removed from the water basin. The time of mice spent in target quadrant (platform previously located) and the number of mice crossing the target was recorded and calculated by the camera coupled with software. Huperzine A was set as the positive control. JWKXS treatment was set for low and high dosages. Values were expressed as Mean ± SEM (*n* = 8). ^#^*p* < 0.05 (compared with sham group), ^∗^*p* < 0.05 (compared with model group).

Morris water maze test is widely used for evaluating cognitive behaviors of animals. Recovering from Aβ injection for 14 days and undergoing 7 days of JWKXS treatment, mice were underwent Morris water maze test and the learning and memory abilities were tested by spatial and probe tests. In spatial test, as results displayed in Figure 1A, model group mice spent more time to find the platform in representative of enhanced latency and distance compared with sham group mice (*p* < 0.05). After JWKXS treatments at two dosages, the latency and distance of mice were decreased significantly compared with model group mice (*p* < 0.05), which was close to the level of sham group, and displayed the strengthened learning abilities. From Figure 1B, in probe test, the decreased levels of time spent in target quadrant and target crossing numbers were also reversed near to the level of sham group with significance (*p* < 0.05) in model mice treated with JWKXS. The tendency of JWKXS was in line with that of huperzine A treatment, which implied that JWKXS possessed the ability of ameliorating cognitive deficits in model mice due to Aβ injection into hippocampus.

**FIGURE 2:**
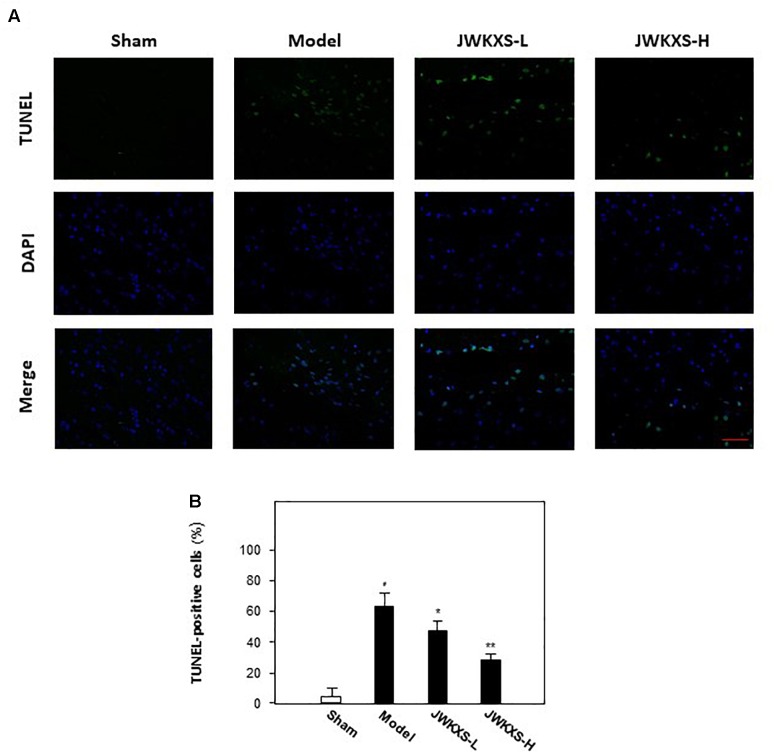
Jia-Wei-Kai-Xin-San treatment decreased apoptosis in hippocampus of Aβ injection mice. **(A)** Apoptosis of cells were determined in hippocampus tissue of mice. TUNEL assay was used to detect apoptotic cells (Scale bar: 100 mm). **(B)** For apoptosis rate determination, the TUNEL positive cells and total cells (DAPI staining) were counted separately and the percentage of TUNEL positive cells to total cells was calculated. Two sections/mouse and three mice were prepared (mean ± SD, ^∗^*p* < 0.05, ^∗∗^*p* < 0.01).

After behavioral tests, TUNEL assays were performed to observe the cell apoptosis of hippocampus tissues in mice brain. Displayed in Figure 2A,B, Aβ injection significantly increased the number of apoptotic cells in hippocampus compared with that of sham group while JWKXS treatment significantly decreased the number of apoptotic cells.

### JWKXS Decreased Aβ Levels and Exerted Anti-oxidation Effect in Hippocampus of Aβ Injection Mice

**FIGURE 3:**
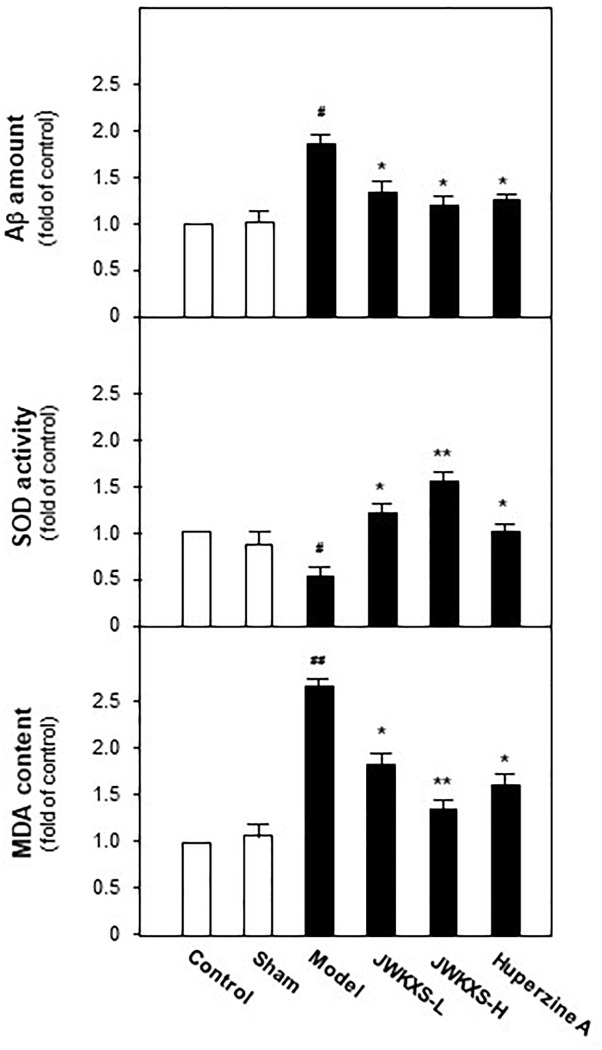
Jia-Wei-Kai-Xin-San treatment decreased Aβ levels, induced SOD activities and decreased MDA levels in hippocampus of Aβ injection mice. Animal group and drug treatment were displayed in Section “Materials and Methods.” After drug treatment, hippocampus tissues were isolated from the brains of animals. Aβ amounts, SOD activities and MDA levels of hippocampus of different group mice were analyzed by described method. Values were expressed in the percentage of control group as the Mean ± SEM (*n* = 8). ^#^*p* < 0.05 (compared with sham group), ^∗^*p* < 0.05, ^∗∗^*p* < 0.01 (compared with model group).

When Morris water maze test finished, all of mice were sacrificed and hippocampus tissues were dissected for biochemical analysis. Aβ levels in hippocampus tissues were determined by ELISA method. Displayed in Figure 3, Aβ levels in hippocampus of model group mice were significantly increased 81% compared with sham group mice (*p* < 0.05) due to the exogenous Aβ injection. After JWKXS treatment, Aβ levels were decreased by ∼31% compared with model group mice (*p* < 0.05), which was in line with the action tendency of positive control huperzine A.

It is well-known Aβ exerts oxidative damages to neuron. Here, effects of JWKXS treatment on SOD activities and MDA levels were evaluated. Shown in Figure 3, Aβ injection significantly decreased activities of SOD by ∼47% compared with sham group (*p* < 0.05). These down-regulated tendencies were successfully reversed by the treatment of two dosages of JWKXS and huperzine A. On the contrary, MDA levels increased by ∼175% in model group compared with sham group (*p* < 0.05) while significantly decreased by ∼47% in JXKXS treatment group compared with model group (*p* < 0.05). Among two dosages of JWKXS, high dosage JWKXS increased activities of SOD by ∼3 folds while decreased MDA levels ∼50% compared with model group (*p* < 0.01), which showed the better anti-oxidative effect.

### JWKXS Treatment Restored Acetylcholine Level, Inhibited Acetylcholinesterase Activities and Increased Choline Acetyltransferase Activities in Hippocampus of Aβ Injection Mice

**FIGURE 4:**
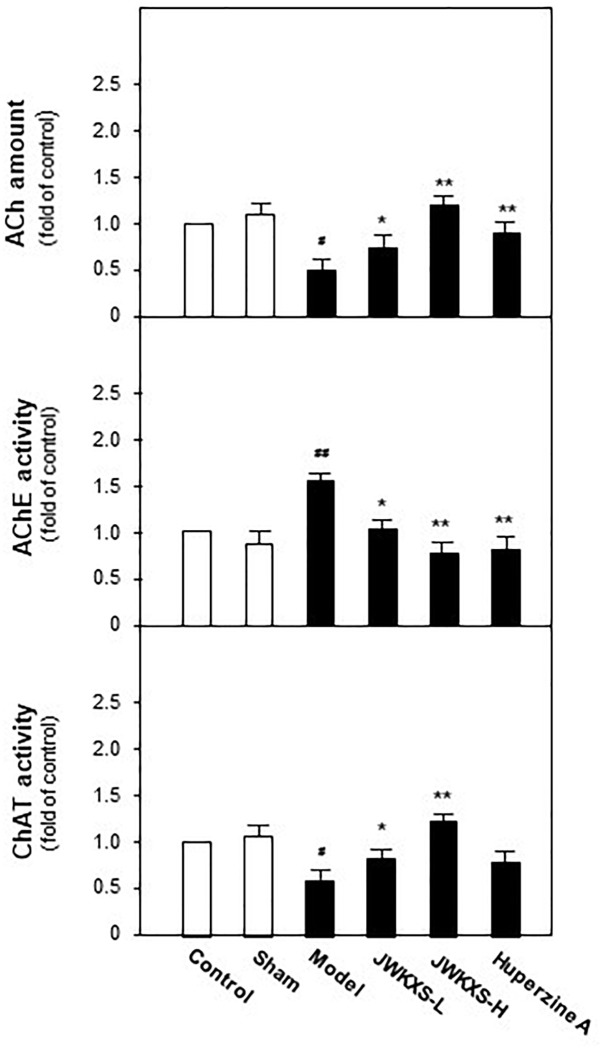
Jia-Wei-Kai-Xin-San treatment increased acetylcholine levels, inhibited AChE activities and induced ChAT activities in hippocampus of Aβ injection mice. Animal group and drug treatment were same as that in Figure 2. After drug treatment, hippocampus tissues were isolated from the brains of animals. The amounts of acetylcholine (ACh) and activities of acetylcholinesterase (AChE) and acetyltransferase (ChAT) were analyzed by described method. Values are expressed in the percentage of control group as the Mean ± SEM (*n* = 8). ^#^*p* < 0.05 (compared with sham group), ^∗^*p* < 0.05, ^∗∗^*p* < 0.01 (compared with model group).

Since cholinergic neurons play a pivotal role in learning and memory, we determined acetylcholine levels, AChE and acetyltransferase activities in hippocampus tissues of all mice. Shown in Figure 4, Aβ injection significantly decreased acetylcholine level by 67% compared with sham group (*p* < 0.05). This decreased tendency was successfully reversed by JWKXS treatment. JWKXS treatment at high dosage increased acetylcholine level by 2.7 folds compared with model group (*p* < 0.01) and low dosage also exerted obvious effect (compared with model group, *p* < 0.05). In parallel, shown in Figure 4, activities of AChE significantly increased by 69% and activities of ChAT significantly decreased by 42% compared with model group (*p* < 0.01) due to Aβ injection. Two dosages of JWKXS treatment decreased AChE activities while increased ChAT activities and the high dosage showed the better effect compared with model group. The action tendency was similar to treatment of well-known AChE inhibitor huperzine A. Since AChE accounts for acetylcholine while ChAT for synthesis, JWKXS up-regulated acetylcholine level by inhibiting hydrolase and increasing synthesis of acetylcholine. Compared with Huperzine A, JWKXS exerted no significant difference in Ach level and AChE inhibition. However, JWKXS was superior to huperzine A in enhancing ChAT activities (*p* < 0.05).

### JWKXS Treatment Decreased Aβ Level via Regulating Genes Related to Aβ Synthesis, Transportation, and Degradation in Hippocampus of Aβ Injection Mice

Based on the findings of decreased level of Aβ in hippocampus of Aβ-injected mice, we further determined mRNA expressions of genes related to Aβ synthesis, degradation and transportation. The determined genes are categorized into three groups: APP, β-secretase 1 (BACE1), Presenilin-1 (PS-1), Presenilin-2 (PS-2) accounting for Aβ synthesis; receptor for advanced glycation end products (RAGE), low density lipoprotein receptor-related protein 1 (LRP1) for Aβ transportation; insulin degrading enzyme (IDE) and neutral endopeptidase (NEP) for Aβ degradation.

**FIGURE 5:**
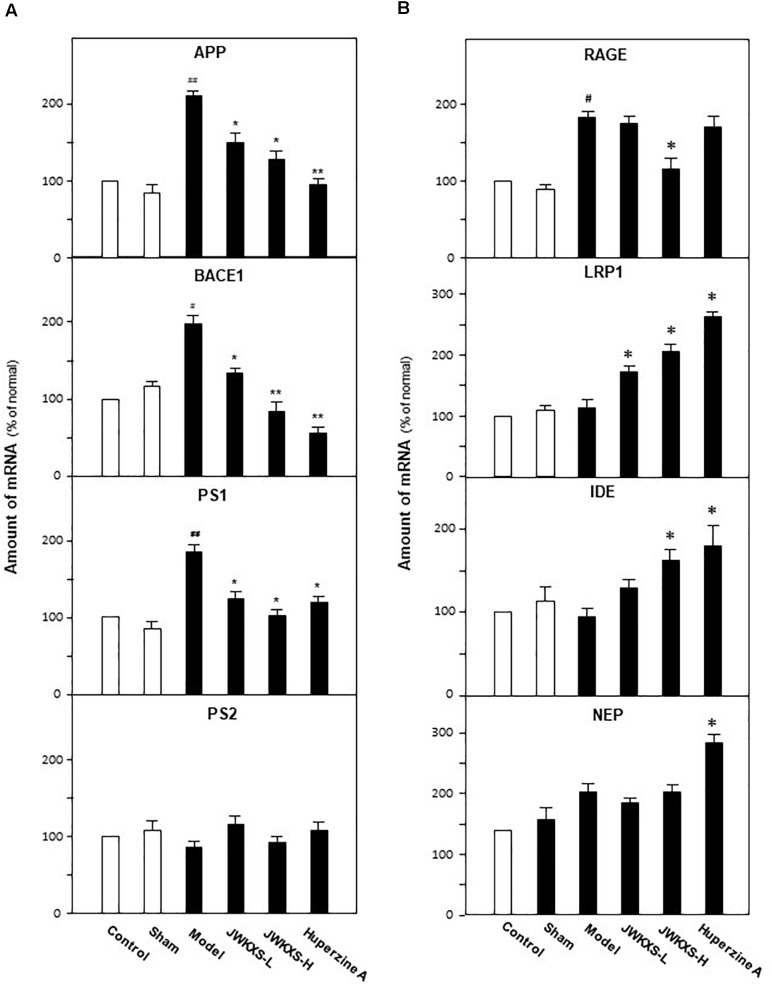
Jia-Wei-Kai-Xin-San treatment modulated mRNA expression of genes relating to Aβ synthesis, transportation and degradation in hippocampus of Aβ injection mice. **(A)** Animal group and drug treatment were same as that in Figure 2. The mRNA expression of genes relating to Aβ synthesis (PS-1, PS-2, APP, BACE1) in hippocampus were analyzed by quantitative PCR. **(B)** The mRNA expression of genes relating to Aβ transportation (RAGE, LRP1) and degradation (IDE, NEP) in hippocampus were analyzed by quantitative PCR. Values were expressed in the percentage of control group as the Mean ± SEM (*n* = 8). ^#^*p* < 0.05, ^##^*p* < 0.01 (compared with sham group); ^∗^*p* < 0.05, ^∗∗∗^*p* < 0.01 (compared with model group).

Shown in Figure 5A, Aβ injection significantly increased the mRNA levels of APP, BACE1, and PS1 in hippocampus of model group compared with sham group (132% for APP, *p* < 0.01; 72% for BACE1, *p* < 0.05; 102% for PS1, *p* < 0.01). Both low and high dosages of JWKXS treatment could down-regulated the mRNA expressions of these Aβ synthesis relating genes compared with model group. High dosages showed better tendency in decreasing mRNA expressions of APP (41%, *p* < 0.05), BACE1 (57%, *p* < 0.01), and PS1 (42%, *p* < 0.05) compared with model group, though no obvious differences found between low and high dosage group according to statistical analysis. Among the genes modulating Aβ synthesis, no matter Aβ injection or JWKXS treatment showed no obvious effects on PS2 expression in our study.

For genes relating Aβ transportation, RAGE accounting for transporting Aβ from blood to brain while LRP1 discharging Aβ from brain to blood. Shown in Figure 5B, Aβ injection significantly increased RAGE expression by 91% (*p* < 0.05) while exerted no obvious effect on LRP1 compared with sham group. Meanwhile, JWXKS treatment at low dosage showed no obvious effect on RAGE expression but increased LRP1 expression by 37% (*p* < 0.05) compared with model group. Comparatively, JWXKS treatment at high dosage significantly decreased RAGE expressions by 41% (*p* < 0.05) and increased LRP1 expressions by 92% compared with model group (*p* < 0.01). Huperzine A also showed on obvious effect on RAGE expressions but increased LRP1 expressions significantly.

As genes relating with two Aβ transportation, Aβ injection exerted on obvious effects on mRNA expressions of IDE and NEP compared with sham group. In parallel, JWXKS treatment at low dosage showed no obvious effect both on IDE and NEP expressions. JWXKS treatment at high dosage significantly increased IDE expressions by 63% but also exerted no obvious effect on NEP expressions compared with sham group (*p* < 0.01). Comparatively, huperzine A showed obvious effect in increasing IDE and NEP expressions compared with model group (*p* < 0.05).

**FIGURE 6:**
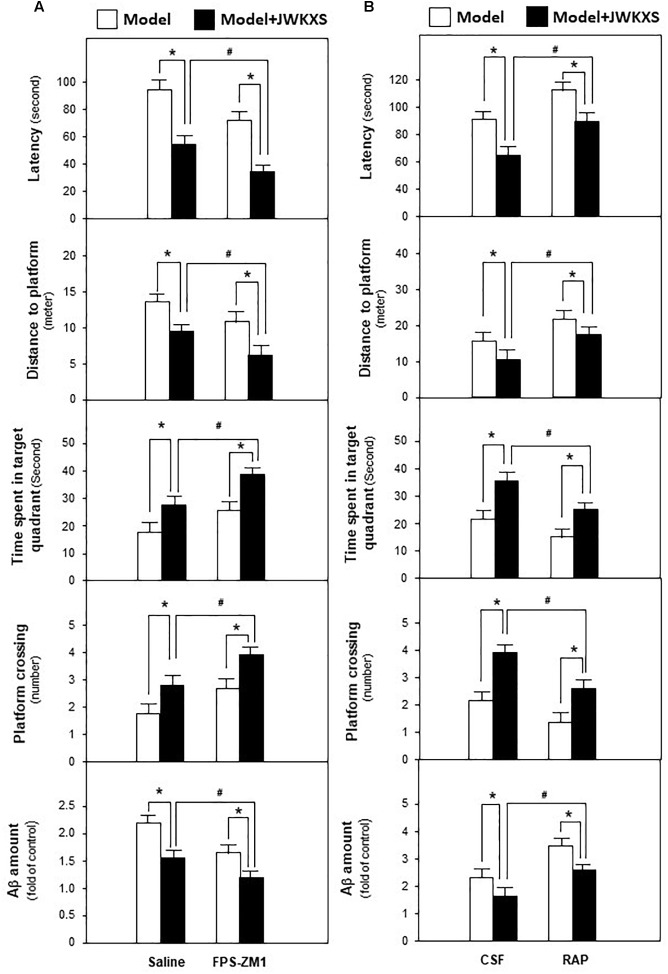
Jia-Wei-Kai-Xin-San treatment modulated proteins relating to Aβ transportation in hippocampus of Aβ injection mice. **(A)** One group of Aβ injection mice were treated with RAGE antagonist FPS-ZM1 and JWKXS (high dosage) simultaneously. JWKXS was orally treated for 7 days and FPS-ZM1 (dissolved in saline) was injected for 3 days. Then, behavioral tests were carried out and Aβ levels were determined. The Aβ injection mice only injected with saline and treated with JWKXS were applied for contrast. **(B)** Aβ injection mice were treated with LRP1 antagonist RAP and JWKXS (high dosage) simultaneously. JWKXS was orally treated for 7 days and RAP (dissolved in artificial CSF) was injected for 3 days. Then, behavioral tests were carried out and Aβ levels were determined. The Aβ injection mice only injected with CSF and treated with JWKXS were applied for contrast. Values are expressed in the percentage of sham group, as Mean ± SEM (*n* = 5). ^∗^*p* < 0.05, ^#^*p* < 0.05.

Based on mRNA expression results, RAGE antagonist FPS-ZM1 and LRP1 antagonist RAP, were applied to determine whether JWKXS improved cognitive deficits by regulating Aβ transportation via RAGE and LRP1. JWKXS at high dosage, exerting most profound improving learning and memory effect, was selected for study. From Figure 6A, the ameliorated cognitive deficits symptoms in JWKXS treatment group, in presence of decreased latency and distances to platform and increased time in target quadrant and platform crossing number, were further improved by the simultaneous FPS-ZM1 treatment. In parallel, FPS-ZM1 plus JWKXS also decreased Aβ levels in hippocampus compared with single JWKXS treatment. On the contrary, displayed in Figure 6B, simultaneous treatment of RAP and JWKXS increased latency and distance to platform and decreased time in target quadrant and platform crossing number compared with single JWKXS treatment. In parallel, RAP treatment also increased Aβ levels in hippocampus.

Based on these phenomena, JWKXS decreased Aβ levels by decreasing Aβ synthesis, accelerating Aβ discharge and degrading Ab in hippocampus.

### JWKXS Treatment Up-Regulated Neurotrophic Factor Levels in Hippocampus of Aβ Injection Mice

**FIGURE 7:**
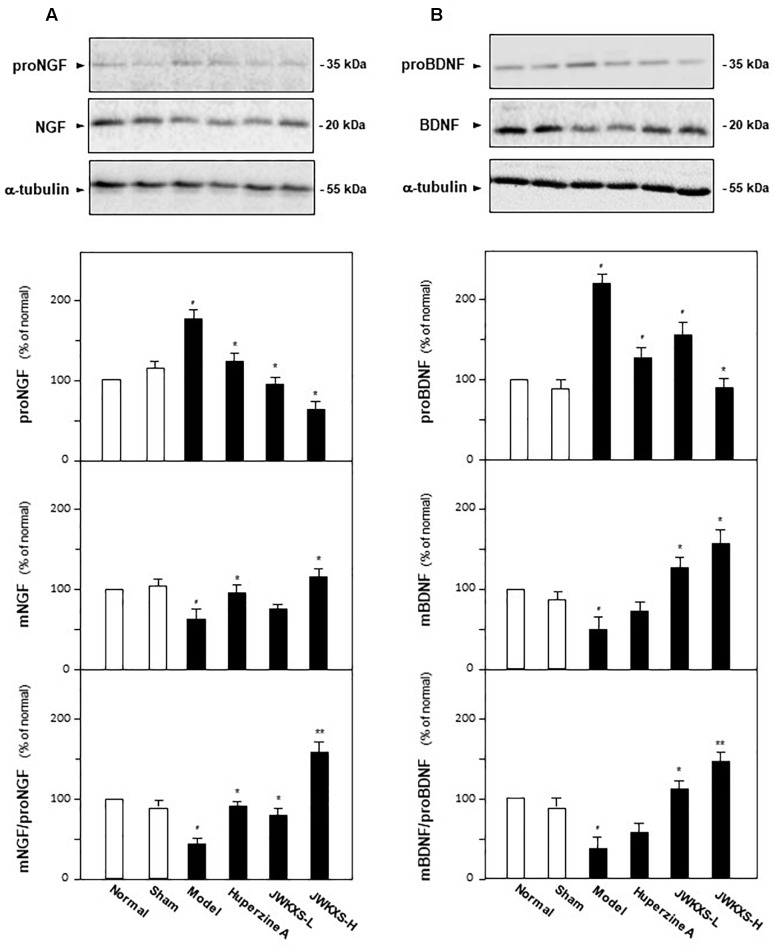
Jia-Wei-Kai-Xin-San treatment induced transformation of neurotrophic factors precursors into mature forms in hippocampus of Aβ injection mice. **(A)** The treatment of JWKXS and huperzine A in the mice were same as that in Figure 1. The hippocampus tissues were collected after 1-week treatment and the expressions of proNGF and NGF were analyzed by western blot analysis and ELISA kits. Afterward, the ratio of mNGF to proNGF was calculated. **(B)** Expressions of proBDNF and BDNF were determined and the ratio of BDNF to proBDNF was calculated as same as that of NGF. Comparisons between groups were carried out by a one-way ANOVA followed by a *post hoc* Bonferroni test. Values were expressed in the percentage of control group as Mean ± SEM (*n* = 8). ^#^*p* < 0.05 (compared with sham group); ^∗^*p* < 0.05, ^∗∗^*p* < 0.01 (compared with model group).

In addition to Aβ, the amounts of two neurotrophic factor closely related to AD, i.e., NGF and BDNF were determined in hippocampus of different groups of mice by western blotting method and ELISA method. Shown in Figure 7, Aβ injection significantly increased proNGF expression by 65% (*p* < 0.05) and decreased mature NGF expression by 35% (*p* < 0.05) compared with sham group. JWXKS treatment both at low and high dosage exerted obvious effects on down-regulation of proNGF and up-regulation of mature NGF. JWXKS treatment at high dosage decreased proNGF expression at 67% (*p* < 0.05) while increased mature NGF expression by 47% (*p* < 0.05) compared with model group. By calculation of the expression ratio of mature NGF to its precursor, Aβ injection inhibited the transformation of proNGF to mNGF by 52% (*p* < 0.05) compared with sham group, and JWKXS treatment reversed this tendency especially in high dosage (increased over two folds of model group, *p* < 0.01). The similar phenomena could also be found in calculation of mBDNF to proBDNF. Based on these phenomena, JWKXS enabled the transformation of precursors of neurotrophic factors to its mature forms and therefore increased the expressions of NGF and BDNF.

### JWKXS Regulated Genes Relating to Synthesis and Degradation of Neurotrophic Factors in Hippocampus of Aβ Injection Mice

Based on the phenomena of JWKXS modulating the expressions and transformation of NGF and BDNF, the mRNA levels of genes relating to processing of neurotrophic factors were determined by quantitative PCR analysis. The critical enzymes could be categorized into two groups: plasminogen, tissue plasminogen activator (tPA), plasminogen activator inhibitor (PAI), and neuroserpin for synthesis while matrix metallopeptidase 9 (MMP-9) and tissue inhibitor of metalloproteinase 1 (TIMP-1) for degradation.

**FIGURE 8:**
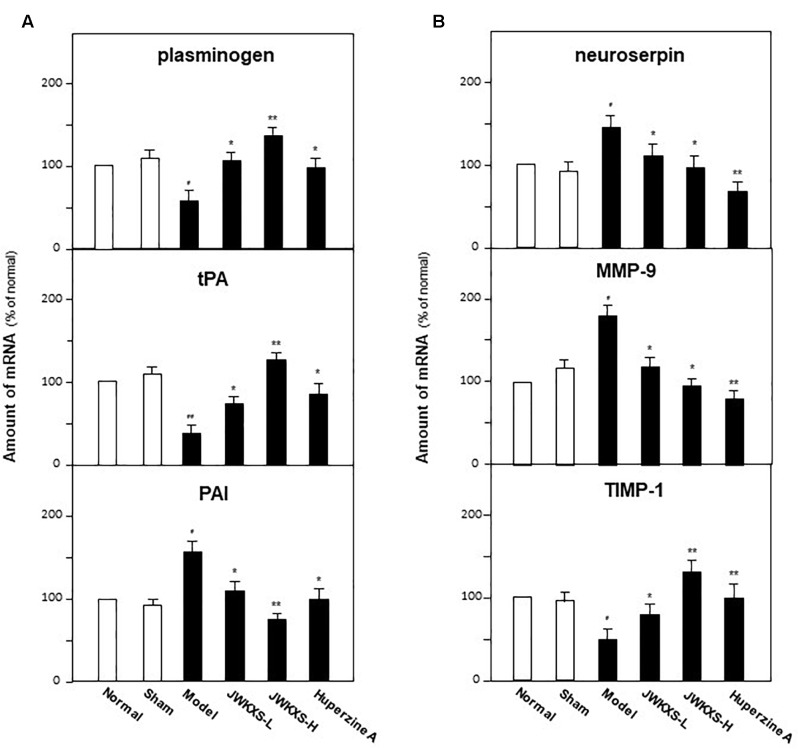
Jia-Wei-Kai-Xin-San modulated mRNA expressions of genes relating to metabolic pathway of neurotrophic factors in hippocampus of Aβ injection mice. **(A)** Animal group and drug treatment were same as that in Figure 2. The mRNA expression of genes relating to neurotrophic factor synthesis (plasminogen, tPA, PAI) in hippocampus of Aβ injection mice were analyzed by quantitative PCR. **(B)** The mRNA expression of genes relating to neurotrophic factor degradation (neuroserpin, MMP-9, TIMP-1) in hippocampus of Aβ injection mice were analyzed by quantitative PCR Values were expressed in the percentage of control group as the Mean ± SEM (*n* = 8). ^#^*p* < 0.05, ^##^*p* < 0.01 (compared with sham group); ^∗^*p* < 0.05, ^∗∗^*p* < 0.01 (compared with model group).

As shown in Figure 8A, Aβ injection significantly decreased the mRNA levels of plasminogen by 47% (*p* < 0.05) and tPA by 65% (*p* < 0.05) while increased PAI by 65% (*p* < 0.05) and neuroserpin by 43% (*p* < 0.05) compared with sham group. Both low and high dosages of JWKXS treatment could up-regulate the mRNA expressions of plasminogen and tPA while down-regulated that of PAI and neuroserpin compared with model group. JWKXS at high dosages showed better tendency in increasing mRNA expressions of plasminogen (136.36%, *p* < 0.05) and tPA (197.62%, *p* < 0.01) while decreasing mRNA expressions of PAI (49.67%, *p* < 0.05) and neuroserpin (33.57%, *p* < 0.05) compared with sham group. JWKXS at high dosage showed better action tendency than low dosage though no obvious differences found between two groups according to statistical analysis. Compared with huperzine A, JWKXS have more potency in transforming precursors of neurotrophic factors into mature form (*p* < 0.05).

For degradation related enzymes, Aβ injection significantly up-regulated mRNA expression of MMP-9 by 42.53% (*p* < 0.05) while down-regulated TIMP-1 expression by 48.12% (*p* < 0.05) in hippocampus compared with sham group. On the contrary, this action tendency led by Aβ injection could be reversed by JWKXS treatment both at low and high dosages. Especially, JWKXS at high dosage decreased MMP-9 mRNA expression by 46.41% (*p* < 0.05) and increased TIMP-1 expression by 168.61% (*p* < 0.01) compared with model group. The action tendency was also similar to huperzine A.

**FIGURE 9:**
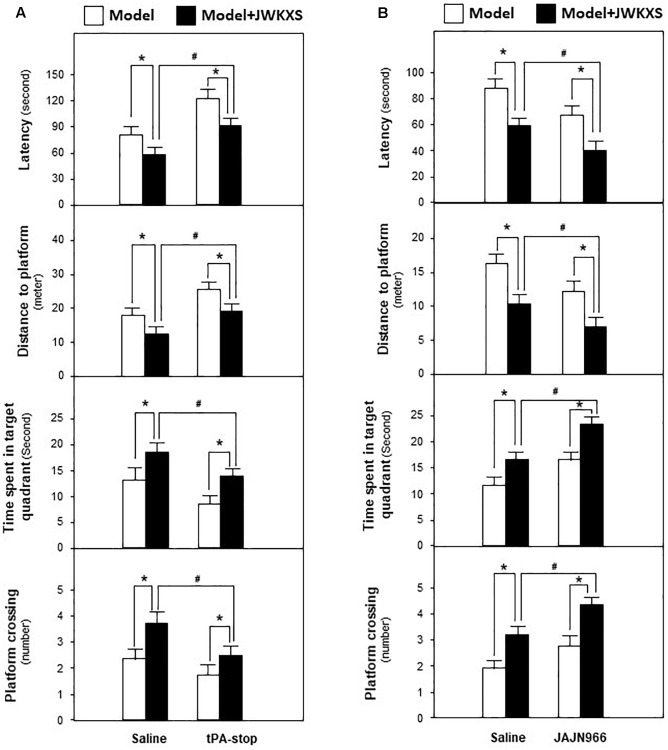
Jia-Wei-Kai-Xin-San modulated proteins relating to neurotrophic factor transformation in hippocampus of Aβ injection mice. **(A)** Aβ injection mice were treated with tPA antagonist tPA-stop and JWKXS (high dosage) simultaneously. JWKXS was orally treated for 7 days and tPA-stop (dissolved in saline) was injected for 3 days. Then, behavioral tests were carried out. The Aβ injection mice only injected with saline and treated with JWKXS were applied for contrast. **(B)** Aβ injection mice were treated with MMP-9 antagonist JAJN966 and JWKXS (high dosage) simultaneously. JWKXS was orally treated for 7 days and JAJN966 (dissolved in saline) was injected for 3 days. Then, behavioral tests were carried out. The Aβ injection mice only injected with saline and treated with JWKXS were applied for contrast. Values are expressed in the percentage of sham group, as Mean ± SEM (*n* = 5). ^∗^*p* < 0.05, ^#^*p* < 0.05.

**FIGURE 10:**
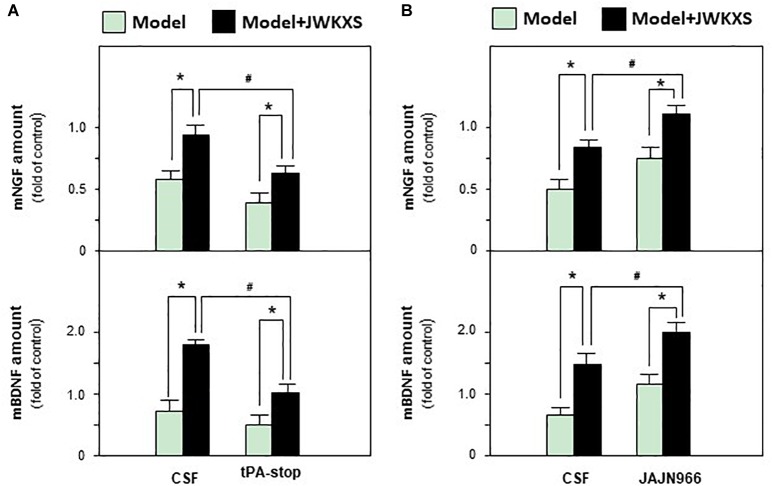
Jia-Wei-Kai-Xin-San treatment modulated neurotrophic factor expressions in hippocampus of antagonist treated Aβ injection mice. **(A)** Drug treatment was same as that in Figure 9A. Then, amount of NGF and BDNF in hippocampus were determined. **(B)** Drug treatment was same as that in Figure 9B. Then, amount of NGF and BDNF in hippocampus were determined. Values are expressed in the percentage of sham group, as Mean ± SEM (*n* = 5). ^∗^*p* < 0.05, ^#^*p* < 0.05.

Based on mRNA expression results, tPA antagonist tPA-stop and MMP-9 antagonist JAJN966, were applied to determine whether JWKXS improved cognitive deficits by regulating neurotrophic factor transformation via tPA and MMP-9. From Figure 9A, the ameliorated cognitive deficits symptoms in JWKXS treatment group were impared by the simultaneous tPA-stop treatment. In parallel, tPA-stop plus JWKXS also decreased NGF and BDNF levels in hippocampus compared with single JWKXS treatment (Shown in Figure 10A). On the contrary, displayed in Figure 9B, simultaneous treatment of JAJN966 and JWKXS improved cognitive deficits by decreasing latency and distance to platform and increasing time in target quadrant and platform crossing number compared with single JWKXS treatment. In parallel, JAJN966 treatment also increased NGF and BDNF levels in hippocampus (Figure 10B).

Based on these data, the increase of neurotrophic factor expressions by JWKXS treatment was related with up-regulation of genes for synthesis and down-regulation genes for degradation.

## Discussion

In current studies, we evaluated the effect of JWKXS on enhancing learning and memory abilities on AD-like animal model and explored the possible action mechanism. Here, we chose Aβ injection hippocampus mice as the animal model. Amyloid beta (Aβ) denoting peptides of 36–43 amino acids is the main component of the amyloid plaques found in brains of AD patients. The peptides derive from the APP, which is cleaved by β-secretase and γ-secretase to produce Aβ. Aβ levels could be increased by enhanced production of Aβ in presence of increased Aβ42/Aβ40 ratio and reduced Aβ clearance. The increased Aβ42 expressions lead to oligomer formation and injure synaptic function. Meanwhile, Aβ42 deposits in parenchyma as diffuse plaques and acquires Aβ fibrils and results in exaggerated inflammatory responses, oxidative stress damages, which leads to neuronal dystrophy and cognitive deficits. This “oligomeropathy” cascade hypothesis is applied for defining AD (Haass and Selkoe, 2007; Blennow et al., 2015). Oligomers or fibrils are all found to induce obvious neuronal injury and synapse loss both *in vivo* and *in vitro*. Aβ exerts oxidative stress on neuron by inducing excessive oxygen radicals, which not only leads to tissue edema and necrosis but also aggravates neuronal excitoxcity by inhibiting glutamate absorption of astrocytes. Besides, Aβ activates microglia releasing cytokines such as IL-1, IL-6 and TNFa, which further worsens the survival surroundings of neuron by inducing neuronal inflammation. Therefore, inhibition of Aβ generation and acceleration of Aβ clearance in brain become hot targets of anti-AD drug development (Scheltens et al., 2016). However, as a complex disease, improvement of neuron injury derived from Aβ should be also taken into account in anti-AD drug development.

In current studies, JWKXS decrease Aβ levels by modulating synthesis, transportation and degradation of Aβ in hippocampus. For synthesis, β-secretas e cleaves APP at the N-terminal site of the Aβ domain, followed by the γ-secretase cleaving at the C-terminal end, thus creating Aβ fragments with diverse lengths such as Aβ40 and Aβ42. BACE 1 and 2 are recognized as the activity center of β-secretas e and BACE 1 seems more important for Aβ production. γ-secretase composed of presenilin, anterior pharynx-defective 1, presenilin enhancer 2 and nicastrin and PS is the catalytic center. We found that JWKXS significantly downregulated mRNA levels of APP, BACE1 and PS1, which implied that JWKXS inhibited production of APP and subsequent cleavage. PS has two mutations named PS1 and PS2 and the mutation of PS1 is far higher than that of PS2 (Ramakrishnan et al., 2017). In parallel, the change of PS1 is easier to be affected than that of PS2. Therefore, we found that JWKXS changed PS1 mRNA level significantly instead of PS2. Besides Aβ synthesis, JWKXS also affected Aβ transportation. Between blood and central nervous system, soluble Aβ removes from blood into brain via RAGE while LRP1 discharges Aβ from brain to blood and these two proteins play important roles in Aβ clearance. Dysfunction of Aβ clearance is reported to account for 99% of AD cases and potent anti-AD drug (Tarasoff-Conway et al., 2015; Wang et al., 2017). Under JWKXS treatment, RAGE mRNA levels were down-regulated while LRP1 were up-regulated. These phenomena implied that JWKXS could inhibit Aβ influx while enable Aβ efflux in brain, which decreased Aβ levels in hippocampus and also validated by application of corresponding inhibitors. In addition to synthesis and transportation, JWKXS also up-regulated mRNA levels of IDE∖, which implied that JWKXS could also affect Aβ degradation. Taken these data together, JWKXS could regulate Aβ via multiple stages, including the production, transportation and degradation, which resulted in the decrease of Aβ in hippocampus. With the decreasing Aβ, we also found the ameliorated oxidative condition in presence of up-regulated SOD activity and down-regulated MDA content in JWKXS treatment group.

In addition to Aβ regulation, JWKXS exerted obvious neuronal nourishment by inducing transformation of precursors of NGF and BDNF into mature form by regulating plasminogen, tPA, PAI and neuroserpin and inhibited degradation of NGF and BDNF by regulating gene MMP-9 and TIMP-1. Survival of cholinergic neuron, the well-known neuronal system in memory formation and maintenance, depends on endogenous mature NGF and BDNF. proNGF converts to mature NGF via coordination of plasminogen, tissue plasminogen activator (tPA), plasmin and neuroserpin. After releasing into extracellular space, a portion of mature NGF binds to receptor tyrosine kinase A (TrkA) to induce neuronal differentiation and some of them degraded by matrix metalloprotease 9 (MMP-9). During degradation of NGF, plasmin also monitors the conversion of proMMP-9 to MMP-9 and tissue inhibitor of metalloproteases 1 (TIMP-1) regulates MMP-9. This regulation loop is not only for NGF but also for BDNF. Contrary to nourish function of mature neurotrophic factors elicited by Trk receptors, proNGF and proBDNF can bind to p75 NTR (neurotrophin receptor) to elicit neuronal apoptosis (Iulita and Cuello, 2014). NGF maturation impairment coupled with increased degradation of mature neurotrophic factors have been validated in AD brains and this dysfunction is also related to Aβ over expressions. On animal studies, Aβ injection in hippocampus significantly increases proNGF levels and MMP-9 activity, which implies that Aβ can trigger NGF metabolic dysfunction even in the absence of tau. Researches also imply that Aβ injures cholinergic neuronal functions in AD patients as well as in transgenic animal models (Davies et al., 1976; Whitehouse et al., 1982; Bell and Claudio, 2006). In our studies, JWKXS treatment significantly up-regulated the mRNA expressions of plasminogen and tPA while down-regulated that of PAI and neuroserpin compared with model group, which implied that JWKXS treatment enabled the transformation of precursors of NGF and BDNF into mature forms and inhibit the degradation of mNGF and mBDNF. Therefore, the levels of mNGF and mBDNF were up-regulated in JWKXS treatment group.

In addition, JWKXS also improved cholinergic neurotransmission function by increasing acetylcholine levels, inhibiting AChE activities and inducing ChAT activities. In center and around of senile plaque, AChE activity is far higher than other brain regions, which enables Aβ to form fiber and enhances neuronal toxicity. Besides, acetylcholine activate cholinergic M1 and M3 receptors can stimulate α-secretase cleavage of APP, which exerts neuronal protection effect by producing APP-releasing soluble APPα (APPS) and C-terminal intracellular fragment (C83) and avoiding producing Aβ. In AD brain, shortage of NGF due to unhealthy stage of cholinergic cannot produce and release sufficient acetylcholine to activate cholinergic receptors, which favors β-secretas e cleavage of APP and increases generation of neurotoxic Aβ (Cuello, 2007). Therefore, the down-regulation of Aβ and up-regulation of neurotrophic factors by JWKXS also benefit for the improvement of cholinergic neuronal transmission.

Right now, U.S. Food and Drug Administration (FDA) has approved five drugs for AD treatment. They are rivastigmine, galantamine, donepezil, memantine, and tacrine. However, tarcrine has been discontinued by FDA for its severe hepatotoxicity. Apart from memantine acting as NMDA receptor antagonist in reducing neuron excitotoxicity, other drugs all target inhibiting AChE activity and restoring acetylcholine reservoir. Huperzine A derived from natural products, a potent AChE inhibitor, is also approved by China Food and Drug Administration (CFDA) for treating AD. In addition to AChE inhibition, huperzine A is also reported to protect neuron from Aβ-induced oxidative injury, antagonizing NMDA receptors targets as well as up-regulate neurotrophic factors (Tang et al., 2005). Similar to huperzine A, JWKXS also could up-regulate Ach level, inhibit AChE activity and up-regulate neurotrophic factor expression but the action targets are different. For Ach up-regulation, JWKXS not only inhibited AChE activity but also benefited for Ach synthesis by inducing ChAT activity while huperzine A showed no obvious effect on ChAT activity. For Aβ regulation, huperzine A showed no obvious effect on RAGE expression while JWKXS exerted no significant effect on Aβ degradation enzyme NEP, which also implied the possible different action target between JWKXS and huperzine A.

However, AChE inhibitors including huperzine A should be used cautiously for AD patients with cardiovascular disease, asthma, and intestinal obstruction. Besides, memantine not only leads to confusion, dizziness, insomnia, agitation and hallucinations, but also induces reversible neurological impairment in people with multiple sclerosis (Scheltens et al., 2016). For JWKXS, these mentioned contraindications and side effects have not been reported in clinic.

According to composition of JWKXS, most of herbs and chemicals have been validated to ameliorate pathological stages of AD. Ginseng Radix and Rhizome is widely applied for treatment of neurodegeneration diseases including AD (Wang et al., 2016). Ginsenosides ameliorate AD-like symptoms via regulating synaptic plasticity, exerting neuroprotection by anti-inflammation and decreasing Aβ production (Kim et al., 2013). Among ginsenosides, Rg_1_ improves spatial learning and memory by reducing Aβ levels in cerebral cortex and hippocampus of AD transgenic model mice and protecting cholinergic neurons from multiple neuronal injuries (Fang et al., 2012). Ginsenoside Rb_1_ decreases Aβ_25-35_ induced tau hyperphosphorylation in primary cultured cortical neurons and the Ca^2+^-calpain-CDK5 signal pathway might be a potential involved pathway (Qian et al., 2009). Polygalae Radix is also a well-known herb applied for treating neurodegeneration and psychiatric disorders and many of the derived compounds are reported to exert profound neuronal bioactivity both on *in vivo* and *in vitro* models (Park et al., 2002; Ikeya et al., 2004; Naito and Tohda, 2006; Sun et al., 2007; Li et al., 2008; Hong et al., 2017). Tenuifolin derived from Polygalae Radix inhibited Aβ secretion *in vitro* and polygalaxanthone III and 3, 6′-disinapoyl sucrose derived from same herb can protect neuronal cells from glutamate and H_2_O_2_ injuries (Li et al., 2008; Hu et al., 2014). For Acori Tatarinowii Rhizoma, recent studies revealed that the herbal extracts and related compounds possess anti-fibrillar amyloid plaques, anti-tau phosphorylation, anti-inflammatory, neuronal protection and neuronal nourish effects, which are highly connected to AD-related biological processes and organs (Lam et al., 2017a,b; Song et al., 2018). α- and β-asarone derived from can improve learning and memory by reducing AChE and Aβ levels and inducing neuronal stem cell proliferation via regulating ERK kinase (Mao et al., 2015; Deng et al., 2016). For Poria, modern researches mainly focus on its anti-tumor activity related with immune regulation (Li et al., 2019). In addition, Poria extract promotes hippocampal long-term potentiation *in vivo* and three compounds poricoic acid A, poricoic acid B, and dehydrotumulosic acid possess the anti-inflammatory function (Smriga et al., 1995; Lee et al., 2016). Polysaccharides derived from Poria also exert anti-depressive and immunosuppressive effects (Li et al., 2019). Schisandra Chinensis Fructus is also widely used for treating neurological diseases and its major compounds lignans can protect the neuronal cell from damage and rescue the injured cognitive performances (Sowndhararajan et al., 2018; Zhang et al., 2018). Among lignans, deoxyschizandrin and schizandrin B possess anti-oxidant and anti-inflammation functions and improve memory deficit on Aβ_1-42_ and scopolamine induced dementia-like rats (Giridharan et al., 2011, 2012; Hu et al., 2012). Gardeniae Fructus is usually applied for treatment of brain aging and vascular aging in TCM. Geniposide, genipin, and crocin fulfill neuroprotective function by rescuing mitochondrion dysfunction, inhibiting apoptosis and exerting anti-inflammatory function (Xiao et al., 2017; Lv et al., 2018). Geniposide inhibits synapse loss and protects neuron from damages of oxidative stress, Aβ and pro-inflammatory cytokines and even regulate autophagy (Liu et al., 2015; Zhang et al., 2019). Petrol fraction and ethyl acetate fraction of Curcumae Radix has been found to possess anti-depressive function (Zhou et al., 2016). Though the direct effect of Ophiopogon japonicas on AD is seldom reported, its major constituents saponins and polysaccharides are reported to possess obvious anti-oxidation and anti-inflammation effects (Chen et al., 2016) Therefore, the function of JWKXS on improving memory and learning deficits might be the synthetic action of multiple compounds derived from each medicinal herbs. In our previous pharmacokinetics studies of JWKXS and other research group studies, most of these compounds also could be detected in plasma of rats (Shi et al., 2018). Based on these research results, we selected a series of chemical compound with definite neuronal activities to develop the quality control method of JWKXS and optimized the preparation procedures, which set a consolidated basis for the drug development (Zhu et al., 2010; Cheng and Tsai, 2016; Qian et al., 2018).

To further discover the action mechanism of JWKXS, we also carried out the mRNA gene chip analysis of mice total brain. According to GO analysis results based on the varied gene expression comparison between model group and JWKXS treatment, disclosed biological process on related to neuronal development include facial and cranial nerve morphogenesis and development, rhombomere development, nerve development and synapse maturation, in which neurotrophic factor regulation plays an important role definitely. Besides, regulation of glutamate receptor signaling pathway and negative regulation of immune system were also listed in the top biological process. In parallel to biological process, significant enriched pathways disclosed by six databases including KEGG, Reactome, PID and so on, also closely related to neuronal development (MAPK targets/nuclear events mediated by MAP kinases, gastrin-CREB signaling pathway via PKC and MAPK, G alpha (q) signaling events), neuron transmission (choline metabolism in cancer; phenylalanine, tyrosine and tryptophan biosynthesis, serotonin receptors) and inflammation regulation (JAK/STAT signaling pathway, T cell receptor signaling pathway, interferon–gamma signaling pathway). Especially, JAK/STAT signaling pathway was both enriched by KEGG and Panther pathway databases, which implied that neuronal inflammation was an important regulation stage in JWKXS exerting anti-AD effect. Therefore, we will further disclose the relationship between effects of JWKXS on improving memory deficits and anti-inflammation and the corresponding regulation network (Paper in review). In addition, the Aβ_1-42_ used in current studies are fibrils created by incubating Aβ_1-42_ at 37°C for a week. This incubation procedures has been accepted in several publications and the formed Aβ fibrils has been applied for developing AD-like animal since Aβ fibrils used to be regarded as the leading cause of AD (Xian et al., 2014a; Shi et al., 2015; Zeng et al., 2016; Tian et al., 2018). In accordance with these reports, Aβ fibrils injection mice exerted cognitive deficits and neuronal injuries in our studies. Therefore, we used Aβ fibrils injection to create AD-like animal model based on these reports. Since Aβ oligomer has been more regarded as the major injury form than Aβ fibrils in brain of AD patients, we will further test JWKXS on Aβ oligomer injection animal model to confirm the efficacy of JWKXS on neuronal protection.

## Conclusion

Jia-Wei-Kai-Xin-San could alleviate cognitive deficits in hippocampus Aβ-injection mice. This function was accounted by decreasing Aβ levels and inducing Aβ efflux, restoring cholinergic neuron normal function and increasing the expression of neurotrophic factors in hippocampus. This herbal extract has been developed into granules and chemically standardized, and therefore which could serve as alternative medicine for patients suffering from AD.

## Author Contributions

YZ, DQ, and J-AD designed the experiments. YS, CC, ML, MQ, RH, and ZZ preformed the experiments including animal model development and behavioral tests. YS and CC contributed to preparation of JWKXS granules and chemical standardization. ZH contributed to writing of introduction and discussion. YZ and YS wrote the main manuscript text. All authors reviewed the manuscript.

## Conflict of Interest Statement

The authors declare that the research was conducted in the absence of any commercial or financial relationships that could be construed as a potential conflict of interest.

## Supplementary Material

Figure S1 Information of components in JWKXS formula. **(A)** Representative figures of herbs in KXS. Bar = 1 cm. **(B)** List of botanical, herbal, and Chinese name of the corresponding herb in JWKXS with its voucher number.

Figure S2 Chemical fingerprint chromatograms of JWKXS formula. **(A)** Fingerprint chromatograms of JWKXS formulae were made by HPLC-DAD at wavelength of 330 nm. The identification of geniposide (1), polygalaxanthone III (2), 3, 6′-disinapoyl sucrose (3), deoxyschizandrin (4), β-asarone (5), and α-asarone (6) were shown in the chromatogram. **(B)** Fingerprint chromatograms of KXS were made by HPLC-ELSD method. The identification of ginsenoside Rg_1_ (7), Rb_1_ (8), and Ophiopogonin B (9) were shown in the chromatogram. Representative chromatograms are shown, *n* = 3.
