# Supplementary figures and images for: Jia-Wei-Kai-Xin-San, an Herbal Medicine Formula, Ameliorates Cognitive Deficits via Modulating Metabolism of Beta Amyloid Protein and Neurotrophic Factors in Hippocampus of Aβ1-42 Induced Cognitive Deficit Mice
Source: Front Pharmacol. 2019 Mar 19;10:258. doi: 10.3389/fphar.2019.00258 (PMC6433786; doi:10.3389/fphar.2019.00258)

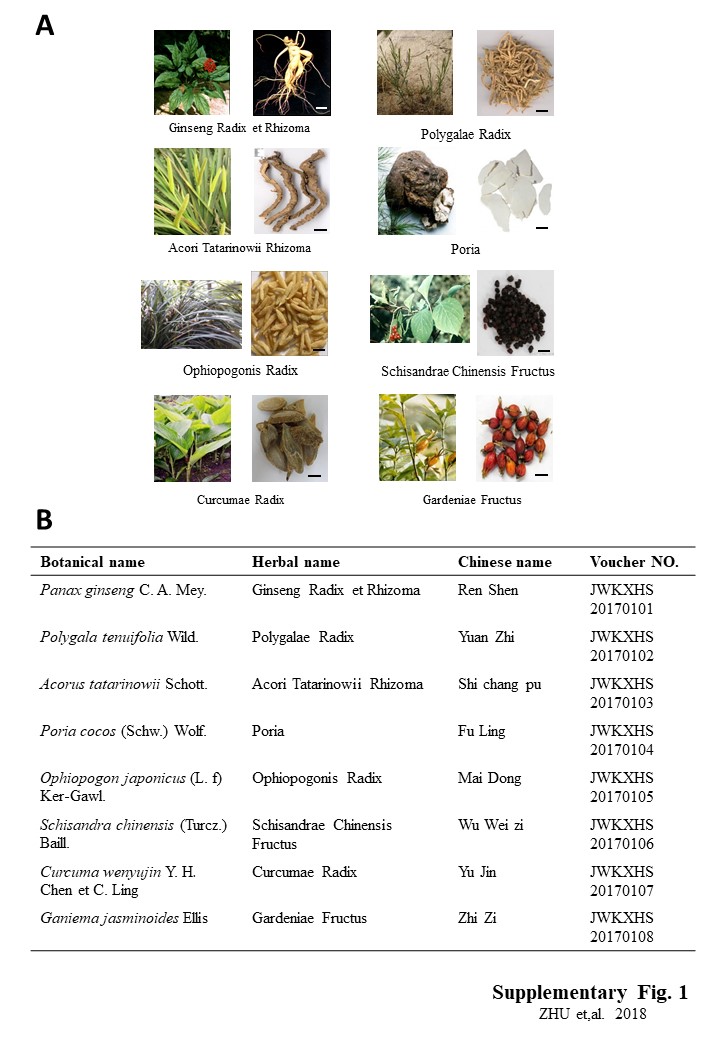

Supplement: Figure S1 — Information of components in JWKXS formula. (A) Representative figures of herbs in KXS. Bar = 1 cm. (B) List of botanical, herbal, and Chinese name of the corresponding herb in JWKXS with its voucher number. [file Image_1.JPEG]

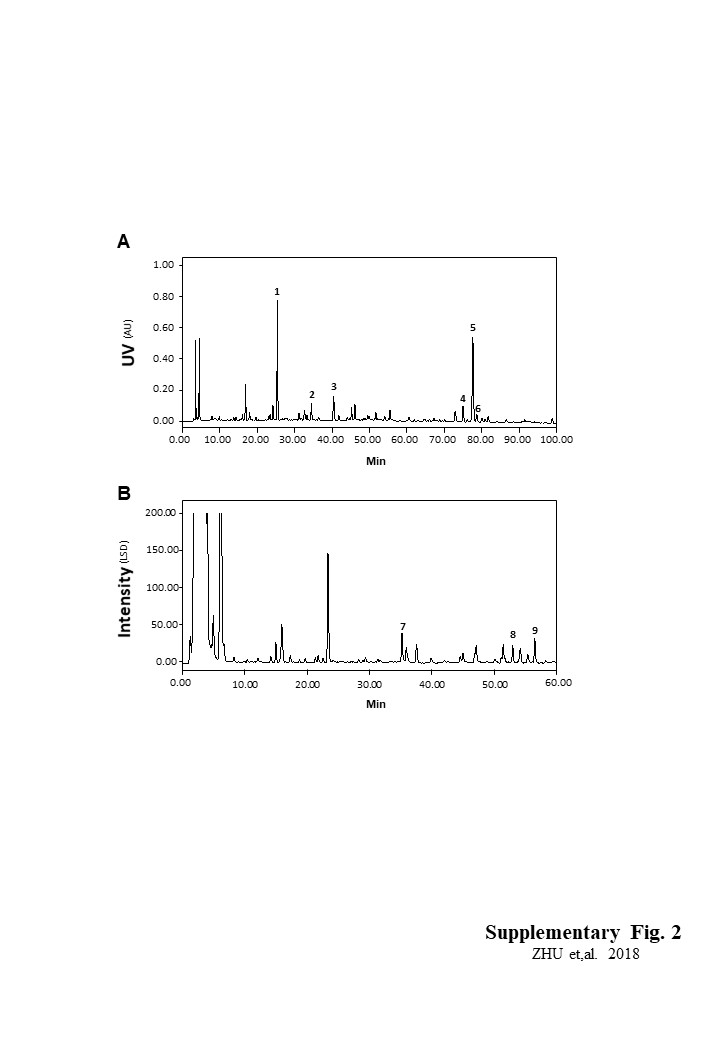

Supplement: Figure S2 — Chemical fingerprint chromatograms of JWKXS formula. (A) Fingerprint chromatograms of JWKXS formulae were made by HPLC-DAD at wavelength of 330 nm. The identification of geniposide (1), polygalaxanthone III (2), 3, 6′-disinapoyl sucrose (3), deoxyschizandrin (4), β-asarone (5), and α-asarone (6) were shown in the chromatogram. (B) Fingerprint chromatograms of KXS were made by HPLC-ELSD method. The identification of ginsenoside Rg1 (7), Rb1 (8), and Ophiopogonin B (9) were shown in the chromatogram. Representative chromatograms are shown, n = 3. [file Image_2.JPEG]
